# Perspectives on Screening Winter-Flood-Tolerant Woody Species in the Riparian Protection Forests of the Three Gorges Reservoir

**DOI:** 10.1371/journal.pone.0108725

**Published:** 2014-09-29

**Authors:** Fan Yang, Yong Wang, Zhulong Chan

**Affiliations:** Key Laboratory of Plant Germplasm Enhancement and Specialty Agriculture, Wuhan Botanical Garden, Chinese Academy of Sciences, Wuhan, Hubei, PR China; CAS, China

## Abstract

The establishment of riparian protection forests in the Three Gorges Reservoir (TGR) is an ideal measure to cope with the eco-environmental problems of the water-level fluctuation zone (WLFZ). Thus, the information for screening winter-flood-tolerant woody plant species is useful for the recovery and re-establishment of the riparian protection forests in the TGR WLFZ. Therefore, we discussed the possibilities of constructing and popularizing riparian protection forests in the TGR WLFZ from several aspects, including the woody plant species distribution in the WLFZ, the survival rate analyses of suitable candidate woody species under controlled flooding conditions, the survival rate investigation of some woody plant species planted in the TGR WLFZ, and the physiological responses of some woody plant species during the recovery stage after winter floods. The results of woody species investigation showed that most woody plant species that existed as annual seedlings in the TGR WLFZ are not suitable candidates for the riparian protection forests. However, arbor species (e.g., *Salix matsudana*, *Populus×canadensis*, *Morus alba*, *Pterocarya stenoptera*, *Taxodium ascendens*, and *Metasequoia glyptostroboides*) and shrub species (e.g., *Salix variegata*, *Distylium chinensis*, *Lycium chinense*, *Myricaria laxiflora*, and *Rosa multiflora*) might be considered suitable candidates for the riparian protection forests in the TGR WLFZ by survival rate analyses under controlled winter flooding conditions, and survival rate investigations of woody plant species planted in the TGR WLFZ, respectively. Physiological analyses showed that *P.×canadensis*, *M. alba*, *L. chinense*, and *S. variegata* could develop specific self-repairing mechanisms to stimulate biomass accumulation and carbohydrate synthesis via the increases in chlorophyll pigments and photosynthesis during recovery after winter floods. Our results suggested these woody plant species could endure the winter flooding stress and recover well, and be used as candidate for the construction of riparian protection forests in the TGR WLFZ.

## Introduction

The portion of the Yangtze River Basin between Chongqing City and Yichang City is known as the Three Gorges Reservoir (TGR) Area (TGRA). The Three Gorges Dam (TGD) was designed to control floods, generate electricity, improve navigation, and create tourism opportunities on the Yangtze River [1]–[3]. The dam was initiated in 1994 and its first impoundment was conducted in 2003 with a water level rising of 60 m above former riverbank of the Yangtze River. The second impoundment was impounded in October 2006 and the water level rose to 156 m. The third impoundment occurred in October 2008 and resulted in a sustained water level at above 170 m for five months. The water was raised to the ultimate planned level of about 175 m above sea level in 2010 [4]–[6]. To operate the TGR at full capacity, the water level of the TGR fluctuates between 145 and 175 m, i.e., 145 m in summer for flood control and emission sediment and 175 m in winter for energy generation. In October, the water level rises gradually to 175 m. By the following January, the water level starts to fall, finally dropping to 145 m in June [3], [5], [7]–[9]. Thus, the peak flows of the TGR occurred during November, December, January, February and March (winter), low flows in June, July, August and September (summer). Therefore, the affecting riparian vegetation factors such as flooding timing, duration, frequency, rate of change, and magnitude [10] in the TGR differed from the natural Yangtze River.

The new hydrological regime, including the reversal of flooding time and the prolonged flooding duration caused by the TGD, dramatically alters the conditions of riparian ecosystems and results in the formation of reservoir water-level fluctuation zone (WLFZ), i.e., the area between the high (175 m) and low (145 m) water levels in the TGR [3], [5]. The WLFZ forms a transitional zone between terrestrial and aquatic ecosystems and serves as an important pathway for exchanging of the fluxes of matter, energy, and information between terrestrial and aquatic ecosystems [7], [11]. In the WLFZ, plants suffered serial submergence stress with durations as long as 210 days at depths of up to 30 m. Few plant species could tolerate such submergence stress. Therefore, the TGR WLFZ has become a disturbed ecosystem, leading to eco-environmental problems, such as biodiversity loss [5], [6], soil erosion, land-use challenges [3], [12], non-point-source pollution [13], [14], nutrient accumulation [15], [16], and heavy metal pollution [17], [18].

Damage to the ecological health and stability of the WLFZ will directly endanger the ecological safety of the whole reservoir area and even the Yangtze River Basin. Riparian protection forests provide numerous ecosystem services including natural scenery improvement, pollutant filtration, wildlife habitat, stream flow mitigation and riverbanks stabilization, and thus maintain the ecological integrity of aquatic and terrestrial ecosystems [19]. However, on one hand, considering the specific hydrological regime in the TGR WLFZ, establishing the riparian protection forest in the whole WLFZ is difficult and even impossible because of prolonged winter flooding durations and navigation disturbances. On the other hand, the riparian protection forest in the upper section of the WFLZ (with an elevation ranging from 170 m to 175 m) can be restored. Therefore, it is important to screen the winter flooding tolerant woody plant species suitable for the construction of riparian protection forests in the TGR WLFZ. For example, mulberry (*Morus alba* L.) had strong adaptation to flooding stress and could survive up to 7 m of flooding in parts of the drawdown zone [20]. The morphological and photosynthetic responses of *P. stenoptera* to a 70 day waterlogging stress showed that this plant species could endure prolonged soil waterlogging with 100% survival rates. Waterlogging stress only slightly affected its photosynthetic apparatus [21]. Compared to unplanted soils, the growth of *P. stenoptera* seedlings significantly increased soil pH value, soil organic matter, total nitrogen, total phosphorus, and total potassium [22]. The seedlings of *Myricaria laxiflora* could survive during the flooding period and recover rapidly after flooding was terminated [23]. The morphological and photosynthetic responses of *Distylium chinense* to simulated autumn and winter floods suggested that this plant species possessed high survival and good recovery growth after a long-term flooding stress [24]. In this paper, we discussed the possibilities of constructing and popularizing riparian protection forests in the TGR WLFZ from several aspects, including the woody plant species distribution in the WLFZ, the survival rate analyses of suitable candidate woody species under controlled flooding conditions (the selection of woody species was determined according to the field survey of pre-dam riparian vegetation in 2001), the survival rate investigation of some woody plant species planted in the TGR WLFZ, and the physiological responses of some woody plant species during the recovery stage after winter floods.

## Materials and Methods

### Experiment 1: Investigation of woody plant species in the TGR WLFZ

A survey on woody plant species distribution was conducted between July 1, 2009 and August 26, 2009. A total of 22 sampling sites (200 m×30 m, surface area) were plotted in the WFLZ along both sides of the Yangtze River in the Zigui, Badong, Wushan, Fengjie, Yunyang, Wanzhou, Zhongxian, Fengdu, Fuling, and Changshou Districts of Chongqing City (the locations of 22 sampling sites were shown in the figure 2 of reference [1]). Few plant species were distributed below the elevation of 160 m, and majority cliffs existed in the WLFZ. Therefore, these 22 large sampling sites were established possibly above the similar elevation (about 160 m). The coordinate data of 22 sampling sites of the water-level-fluctuation zone in the Three Gorges Reservoir in 2009 was shown in Table 1. Plant specimens were collected in the TGR WLFZ. Basic parameters, such as the lowest distributed elevation among different sample sites of each plant species, plant growth age and height were recorded. No specific permissions were required for all of the field studies. The field study did not involve endangered or protected species.

**Table 1:** The coordinate data of 22 sampling sites of the water-level-fluctuation zone in the Three Gorges Reservoir in 2009.

No. sampling site	Sampling site	Longitude (E)	Latitude (N)	Lower elevation (m)	No. of sampling sites	Sampling site	Longitude (E)	Latitude (N)	Lower elevation (m)
**1**	Longxi River in Changshou	107°05′12.2″	29°48′43.6″	162	**12**	Yangtze River in the old location of Yunyang	108°54′09.0″	30°57′24.2″	158
**2**	Changming Port in Changshou	107°01′11.7″	29°47′17.3″	160	**13**	Yangxi River in the old location of Yunynag	108°54′07.4″	30°57′45.8″	160
**3**	Wu River in Fuling	107°23′33.4″	29°40′38.6″	165	**14**	Meixi River in Fengjie	109°31′25.8″	31°03′04.5″	160
**4**	Yangtze River in Fuling	107°24′47.2″	29°44′39.9″	162	**15**	Yangtze River in Fengjie	109°28′03.9″	31°00′21.7″	158
**5**	Yangtze River in Fengdu	107°40′51.0″	29°51′48.3″	164	**16**	Yangtze River in Wushan	109°53′29.0″	31°04′19.8″	160
**6**	Long River in Fengdu	107°44′43.7″	29°52′28.1″	162	**17**	Yangtze River in the Badong	110°23′42.0″	31°02′30.4″	160
**7**	Yangtze River in Zhong Xian	108°03′11.2″	29°18′08.9″	156	**18**	Padang Port in Badong	110°19′30.6″	31°02′31.3″	160
**8**	Xintian Town in Wanzhou	108°23′28.4″	30°41′41.7″	155	**19**	Shennong River in Badong	110°49′27.7″	31°03′40.2″	160
**9**	Wanzhou Port in Wanzhou	108°23′46.7″	30°41′15.1″	160	**20**	Xiangxi River in Zigui	110°40′48.1″	31°03′50.9″	160
**10**	Pengxi River in Yunyang	108°41′16.1″	30°56′54.2″	160	**21**	Lanling River vegetation recovery test base in Zigui	110°55′11.7″	30°51′55.3″	160
**11**	Yangtze River in Yunyang	108°42′48.0″	30°55′06.2″	162	**22**	Vegetation recovery test base in Three Gorges Dam in Zigui	110°59′08.9″	30°51′18.3″	160

### Experiment 2: Survival rate analysis of the candidate woody plant species after controlled winter flooding

The selection of candidate woody plant species were determined the field survey of the pre-dam riparian vegetation of natural Yangtze River in 2001. The screening experiments of candidate woody plant species for the TGR WLFZ were conducted during 2006 to 2011 at Wuhan Botanical Garden, Chinese Academy of Sciences, and Lanling Creek, which is a Yangtze River tributary. Two year-old seedlings of 11 woody plant species from arbor species (e.g., *Salix matsudana*, *Populus×canadensis*, *M. alba*, *P. stenoptera*, *Taxodium ascendens*, and *Metasequoia glyptostroboides*) and shrub species (e.g., *Salix variegata*, *D. chinensis*, *Lycium chinense*, *M. laxiflora*, and *Rosa multiflora*) were transplanted into 30 cm*×*15 cm pot. After a growing season, the branches of all plants were pruned to an identical height, i.e., 40 cm above ground level, and the leaves were removed. According to the the reservoir water level regulation, four submerged depths (1, 5, 15, and 25 m) and three submerged times (90, 150, and 210 days) were arranged as the sub-plot and main plot, respectively. The 1 m-deep submerged experiment was performed in two pools at Wuhan Botanical Garden, and other three submerged experiments (5, 15, and 25 m) were performed at Lanling Creek. For 5, 15, and 25 m submerged experiments, all the submerged pots were hung at the different pneumatic tires according to their individual submerged depths, and all the tires through the wire rope were fixed at the both sides of the river. The submergence treatment was conducted in batches in coordination with the submergence durations since September each year. All submerged plants were reaped simultaneously for analysis in April next year.

### Experiment 3: Investigation of survival rate of some woody plant species planted in the TGR WLFZ

The majority of one-year-old *M. alba* (mulberry) seedlings were planted in March 2010 in the WLFZ of Xiaohe Village, Kai County, Chongqing, Yangtze River tributary, with an elevation ranging from 170 m to 175 m. Their survival rate was more than 95% before the next TGR impoundment. The survival plants were recorded in September 2010, and investigation on the survival rate of *M. Alba* was conducted in May 2011. The majority of seedlings of *S. variegata*, *P.×canadensis*, *T. ascendens*, *M. glyptostroboides*, and *L. chinense* were planted in 2008 in the WLFZ of the Lanling Creek, Yangtze River tributary, with an elevation ranging from 165 m to 175 m. The submergence durations were over 100 days each year since 2010. The survival plants were recorded in September 2011. The investigations on the survival rate of *S. variegata*, *P.×canadensis*, *T. ascendens*, *M. glyptostroboides*, *L. chinense*, and *M. alba* were conducted in May 2012.

### Experiment 4: Dertermination the chlorophyll pigments, photosynthesis,chlorophyll a fluorescence of some woody plant species planted in the TGR WLFZ during the recovery stage after winter flooding

Fresh leaves from the six plants of *S. variegata*, *L. chinense*, *P.×canadensis*, and *M. alba* planted at an elevation of 176 m (used as control) and 172 m of the Lanling Creek WLFZ were collected on April 10, 2012. The collected leaves were frozen in liquid nitrogen immediately for pigment content analyses. The contents of chlorophyll a (Ch *a*), chlorophyll b (Ch *b*), carotenoids were determined according to the procedures of [25]. Anthocyanin content was determined according to the procedures of [26] with a slight modification. The anthocyanin was extracted by 1% HCl–methanol solvent (1∶99, v∶v). Relative content was then determined by the absorbance at 532 nm. To alleviate the effects of chlorophyll, the absorbance of anthocyanin was calculated as follows:
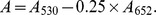



The gas exchange and chlorophyll a fluorescence parameter analyses of *P.×canadensis* and *M. alba* were performed according to the procedures of [25]. The same six cuttings and same leaves were selected and used for gas exchange measurements and chlorophyll fluorescence measurements. The net CO_2_ assimilation rate (*A*), stomatal conductance (*g_s_*), intercellular CO2 concentration (*Ci*) and transpiration (*E*) were measured from 9:00 to 11:30 am on April 9, 2012 with a LI-COR 6400 portable photosynthesis system (LI-COR Inc. Lincoln, Nebr.). The PAR, provided by a 6400-02 LED light source, was set to 1200 µmol·m^−2^·s^−1^. The flow rate of air through the sample chamber was set at 500 µmol·m^−2^·s^−1^, and the leaf temperature and relative humidity was maintained at 25±0.8°C by thermoelectric coolers and 50%, respectively. Chlorophyll fluorescence kinetics parameters were measured with a portable chlorophyll fluorometer PAM 2500 (Walz, Effeltrich, Germany). The leaf samples were placed in darkness for 30 min by covering with dark leaf clip (DLC-8) followed by measurement of minimum fluorescence (Fo) at 250 µmol m^2^s^−1^ PPF and Fm at 2400 µmol m^2^s^−1^ PPF following a saturating pulse of actinic light).

### Statistical analyses

Results were expressed as means ± standard errors (n = 6). SPSS 13.0 software was used for statistical analysis. To assess the statistical significance of the treatment differences, a one-way analysis of variance (ANOVA) followed by Duncan's multiple range test was employed. Differences were considered significant at P<0.05.

## Results

### Woody plant species distribution in the TGR WLFZ

The woody plant species in the TGR WLFZ was obtained from the 22 sampling regions. The results showed that 39 woody plant species including 12 tree species and 27 shrub or sub-shrub species, belonging to 37 genera of 24 families were distributed in the TGR WLFZ (Table 2). Most of these woody species existed as annual seedlings, except for *Sapium sebiferum*, *Ficus tikoua*, *L. chinense*, *M. alba*, *M. australis*, *P.×canadensis*, *S. matsudana*, *R. multiflora*, *S. variegata M. laxiflora*, and *D. chinense*.

**Table 2:** Woody plant species distribution in the TGR WLFZ in 2009.

Species	Elevation (>m)	Plant height (cm)	Life form (Growth age)	Species	Elevation (>m)	Plant height (cm)	Life form (Growth age)
***1 Leguminosae*** a **(4** b **: 4** c **)**				***2Rosaceae*** ** (2: 2)**			
*Bauhinia brachycarpa*	173	60–80	Annual shrub	*Rosa multiflora*	172	20–70	Perennial climbing shrub
*Indigofera pseudotinctoria*	170	100–150	Annual shrub	*Rubus parvifolius*	174	30–50	Annual shrub
*Lespedeza cuneata*	172	60–780	Annual woody vines	***3 Euphorbiaceae*** ** (1: 1)**			
*Robinia pseudoacacia*	168	50–100	Annual tree seedlings	*Sapium sebiferum*	165	50–140	Annual tree seedlings
***4 Moraceae*** ** (3: 4)**				***5 Salicaceae*** ** (2: 3)**			
*Broussonetia papyrifera*	165	30–90	Annual tree seedlings	*Salix matsudana*	168	150–350	Perennial tree
*Cudrania tricuspidata*	171	100–180	Annual shrub	*S. variegata* Franch.	164	40–130	Perennial shrub
*Morus alba*	169	90–450	Perennial arbor	*Populus*×*canadensis*	165	150–500	Perennial tree
*M. australis*	172	150–250	Perennial arbor	***6 Anacardiaceae*** ** (1: 1)**			
***7 Verbenaceae*** ** (1: 2)**				*Rhus chinensis Mill.*	167	20–80	Annual tree seedlings
*Vitex negundo*	168	30–140	Annual shrub	***8 Loganiaceae*** ** (1: 1)**			
*V. negundo* var. *cannabifolia*	168	30–120	Annual shrub	*Buddleja davidii*	170	40–200	Annual shrub
***9 Caprifoliaceae*** ** (1: 1)**				***10 Celastraceae*** ** (1: 1)**			
*Sambucus chinensis*	172	80–130	Annual subshrub	*Maytenus variabilis*	173	20–30	Annual shrub
***11 Liliaceae*** ** (1: 1)**				***13 Rutaceae*** ** (2: 2)**			
*Smilax china*	173	25	Annual shrub	*Phellodendron chinense var.glabriusculum*	170	20–70	Annual tree seedlings
***12 Solanaceae*** ** (3: 3)**				*Zanthoxylum armatum*	172	25–45	Annual shrub
*Lycium chinense*	170	30–90	Perennial Shrub	***15 Coriariaceae*** ** (1: 1)**			
*Solanum surattense*	172	40–70	Annual subshrub	*Coriaria nepalensis*	170	20–30	Annual shrub
*Datura innoxia*	173	40–90	Annual shrub	***16 Tamaricaceae*** ** (1: 1)**			
***14 Hamamelidaceae*** ** (1: 1)**				*Myricaria laxiflora*	172	60–110	Perennial shrub
*Distylium chinense*	166	30–70	Perennial shrub	***18 Sapindaceae*** ** (1: 1)**			
***17 Juglandaceae*** ** (1: 1)**				*Koelreuteria paniculata*	165	20–90	Annual tree seedlings
*Pterocarya stenoptera*	172	100–450	Perennial tree	***20 Malvaceae*** ** (2: 2)**			
***19 Urticaceae*** ** (1: 1)**				*Hibiscus mutabilis*	174	100–140	Annual tree seedlings
*Debregeasia orientalis*	173	40–70	Annual shrub	*Urena lobata*	171	30–60	Annual shrub
**22 Nyssaceae (1: 1)**				**21 Ulmaceae (2: 2)**			
*Camptotheca acuminata*	172	50–150	Annual tree seedlings	*Trema laevigata*	172	30–50	Annual shrub
**24 Araliaceae (1: 1)**				*Ulmus pumila*	173	76	Annual tree seedlings
*Aralia echinocaulis*	173	35	Annual tree seedlings	**23 Linaceae (1: 1)**			
				*Reinwardtia indica*	171	20	Annual shrub

a , family name;

b , the number of genera within each family;

c , the number of species within each family.

### Survival rate analyses of 11 woody plant species after controlled winter flooding

Under 1 m-deep submerged condition, all tested plants could survive after 90 days of submergence. Most of the tested plants can endure 90 days of submergence with a survival rate of more than 80%; *L. chinense* and *M. laxiflora* species possessed a survival rate of more than 50% (Table 3). *S. matsudana*, *P.×canadensis*, *M. alba*, *T. ascendens*, *M. glyptostroboides*, *S. variegata*, *R. multiflora*, and *D. chinensis* could endure 150 days of submergence with survival rates of more than 50%. *S. matsudana*, *T. ascendens*, *M. glyptostroboides*, *S. variegata*, *R. multiflora*, and *D. chinensis* could endure 210 days of submergence with a survival rate of more than 20%.

**Table 3:** Survival rate analyses of 11 woody plant species under controlled flooding conditions.

Plant species	Submergence treatment												
	Submergence depth (m)	1			5			15			25		
	Submergence duration (days)	90	150	210	90	150	210	90	150	210	90	150	210
*Salix variegata*	Number of the test plants	50	50	60	30	30	30	30	30	30	30	30	30
	Number of survival plants	45	37	32	26	18	14	10	6	3	5	3	0
	**Survival rate (%)**	**90**	**74**	**53**	**87**	**60**	**47**	**33**	**20**	**10**	**17**	**10**	**0**
*Distylium chinensis*	Number of the test plants	30	30	28	30	30	30	30	30	30	30	30	26
	Number of survival plants	28	24	15	25	17	10	9	6	0	0	0	0
	**Survival rate (%)**	**93**	**80**	**54**	**83**	**57**	**33**	**30**	**20**	**0**	**0**	**0**	**0**
*Rosa multiflora*	Number of the test plants	10	10	10	10	10	10	10	10	10	10	10	18
	Number of survival plants	8	6	3	8	5	1	2	0	0	0	0	0
	**Survival rate (%)**	**80**	**60**	**30**	**80**	**50**	**10**	**20**	**0**	**0**	**0**	**0**	**0**
*Salix matsudana*	Number of the test plants	90	90	90	30	30	30	30	30	30	30	30	26
	Number of survival plants	75	55	26	25	16	4	5	0	0	0	0	0
	**Survival rate (%)**	**83**	**61**	**29**	**83**	**53**	**13**	**17**	**0**	**0**	**0**	**0**	**0**
*Taxodium ascendens*	Number of the test plants	10	10	10	10	10	10	10	10	10	10	10	10
	Number of survival plants	9	7	4	8	6	3	4	2	0	0	0	0
	**Survival rate (%)**	**90**	**70**	**40**	**80**	**60**	**30**	**40**	**20**	**0**	**0**	**0**	**0**
*Metasequoia glyptostroboides*	Number of the test plants	10	10	10	10	10	10	10	10	10	10	10	10
	Number of survival plants	9	8	3	8	5	2	3	1	0	0	0	0
	**Survival rate (%)**	**90**	**80**	**30**	**80**	**50**	**20**	**30**	**10**	**0**	**0**	**0**	**0**
*Morus alba*	Number of the test plants	20	20	20	25	25	25	25	25	25	25	25	25
	Number of survival plants	16	11	2	18	14	2	3	0	0	0	0	0
	**Survival rate (%)**	**80**	**55**	**10**	**72**	**56**	**8**	**12**	**0**	**0**	**0**	**0**	**0**
*Populus×canadensis*	Number of the test plants	30	30	30	30	30	30	30	30	30	30	30	30
	Number of survival plants	26	17	4	22	12	2	3	0	0	0	0	0
	**Survival rate (%)**	**87**	**57**	**13**	**73**	**40**	**7**	**10**	**0**	**0**	**0**	**0**	**0**
*Pterocarya stenoptera*	Number of the test plants	10	10	10	10	10	10	15	15	15	15	15	15
	Number of survival plants	8	4	1	4	2	0	1	0	0	0	0	0
	**Survival rate (%)**	**80**	**40**	**10**	**40**	**20**	**0**	**7**	**0**	**0**	**0**	**0**	**0**
*Myricaria laxiflora*	Number of the test plants	10	10	10	10	10	10	10	10	10	10	10	10
	Number of survival plants	9	3	1	5	1	0	1	0	0	0	0	0
	**Survival rate (%)**	**90**	**30**	**10**	**50**	**10**	**0**	**10**	**0**	**0**	**0**	**0**	**0**
*Lycium chinense*	Number of the test plants	30	30	30	30	30	30	30	30	30	30	30	30
	Number of survival plants	22	12	2	18	5	3	1	0	0	0	0	0
	**Survival rate (%)**	**73**	**40**	**7**	**60**	**16**	**10**	**3**	**0**	**0**	**0**	**0**	**0**

Under 5 m-deep submerged conditions, all tested plants could survive after 90 days of submergence. Most of the tested plants could endure 90 days of submergence with a survival rate of more than 70%; *P. stenoptera*, *M. laxiflora*, *L. chinense* and *M. laxiflora* species possessed a survival rate of more than 30%. *S. matsudana*, *M. alba*, *T. ascendens*, *M. glyptostroboides*, *S. variegata*, *D. chinensis*, and *R. multiflora* could endure 150-days of submergence with a survival rate of more than 50%. *T. ascendens*, *M. glyptostroboides*, *S. variegata*, and *D. chinensis* could endure 210-days of submergence with a survival rate of more than 20% (Table 3).

Under 15 m-deep submerged conditions, *T. ascendens*, *M. glyptostroboides*, *S. variegata*, and *D. chinensis* could endure 90-days of submergence with a survival rate of more than 25%, and they could survive after 150-days submergence but with a low survival rate. Only few plants of *S. variegata* could endure 210-days of submergence and survive (Table 3).

Under 25 m-deep submerged conditions, only few plants of *S. variegata* could endure 150-days of submergence and survive, and all tested woody species could not survive after 210-days of submergence (Table 3).

### Investigation of survival rate of some woody plant species planted in the TGR WLFZ

Mulberry seedlings planted in the Xiaohe Village WLFZ could survive after one winter flooding season but possessed different survival rates with variations in elevation from 170 m to 175 m. The plants planted in the sloping fields showed higher survival rates than the planted in the paddy field because of the different drainage property of the landform in the growth season. Plants with old branches also showed higher survival rates than plants without branches. Therefore, the survival rate of mulberry was simultaneously determined by several factors, including submergence duration and depth, landform drainage property, and old branch conditions (Table 4). The mulberry in the WLFZ could endure 5 m-depth submergence for over 120 days and form new crowns.

**Table 4:** Survival rate analyses of *M. alba* in 2012 planted in the Xiaohe village WLFZ.

		Elevatation(m)				
		175-174	174-173	173-172	172-171	171-170
Total Area (m^2^)		600	600	400	400	300
Number of the investigated plants		500	500	500	500	500
Submerged Days		≤83	≤95	≤107	≤118	≤127
Average survival rate (%)		75.7	70.9	42.1	30.3	23.4
Survival rate (%)	Sloping field	82.7	71.9	ND	53.5	ND
	Paddy field	70.8	52.9	42.1	28.3	23.4
Survival rate (%)	With old branches	ND	85.9	47.8	ND	ND
	Without old branches	75.7	61.9	32.1	36.3	23.4

ND, not determined.

After four seasonal winter flooding, the survival conditions of *P.×canadensis*, *S. variegata*, *T. ascendens*, *M. glyptostroboides*, and *L. chinense* planted in the Langling Creek WLFZ were detected (Table 5). These five woody plants could endure tremendous winter flooding stress and survive well. *P.×canadensis*, *S. variegata*, *T. ascendens*, and *M. glyptostroboides* possessed equivalent strong survival rates, whereas *L. chinense* had low survival rates. Survival rate was affected by full submersion and invasion. Although some plants died each year for different reasons, including flooding stress, human destruction, ecological competitions, plant diseases, and insect pests, the protection forests of *P.×canadensis*, *T. ascendens*, and *M. glyptostroboides* thrived, specifically the *P.×canadensis* forests. However, the shrub species, including *S. variegata* and *L. chinense*, planted in the WLFZ were seriously threatened by majority weeds such as *Bidens tripartite*, *Bidens bipinnata*, *Xanthium sibiricum*, *Alternanthera philoxeroides*, and *Setaria viridis* because of the dominant plant communities (high height, intensities, and coverage) and rapid growth of such weeds, except for winter flooding stresses.

**Table 5:** Survival rate analyses of five woody plants in 2012 planted in the Langling Creek WLFZ.

The test plant species	Elevatation (m)															
	173				171				169				167			
	Number of test plants	Sub. C.	Inv. W.	Suv. R.	Number of test plants	Sub. C.	Inv. W.	Suv. R.	Number of test plants	Sub. C.	Inv. W.	Suv. R.	Number of test plants	Sub. C.	Inv. W.	Suv. R.
*P.×canadensis*	25	Partial	×	100%	19	Partial	×	100%	20	Partial	×	70%	20	Full	×	30%
*S. variegata*	10	Full	√	100%	10	Full	√	90%	9	Full	√	78%	5	Full	√	60%
*T. ascendens*	25	Partial	×	100%	14	Partial	×	100%	ND				15	Full	×	60%
*M. glyptostroboides*	8	Partial	×	100%	6	Partial	×	100%	ND				6	Full	×	50%
*L. chinense*	20	Full	√	75%	20	Full	√	55%	ND				20	Full	√	15%

Sub. C., submersion conditions (Full submersion, partial submersion); Inv. W., whether invaded by dominant plant communities or not; Sur. R., survival rate; ND, not determined.

### Physiological responses of some woody plant species planted in the TGR WLFZ during the recovery stage after winter flooding

A slight increase in the contents of Ch *a* and Ch *b*, net photosynthetic rate (*A*), stomatal conductance (*gs*), intercellular CO_2_ concentration (*C_i,_*), and transpiration rate (*E*) were found in the submerged *P.×canadensis* during the recovery stage after winter floods compared with the controls. No significant differences on these parameters were found between other submerged plants and the controls. However, the content of carotenoids was significantly increased by flooding stress. The maximum efficiency of PSII (Fv/Fm) in the submerged *P.×canadensis* during the recovery stage after winter floods maintained higher levels in comparison to the controls, whereas other chlorophyll fluorescence parameters were not significantly affected by flooding stress (Table 6).

**Table 6:** Physiological responses of *P.×canadensis* planted in the TGR WLFZ during the recovery stage after winter flooding.

	Ch *a* (mg/g Fw)	Ch *b* (mg/g Fw)	Carotenoids (mg/g Fw)	*A*	*g_s_*	*Ci*	*E*	Fv/Fm	Yield	Y(NPQ)	Y(NO)	qN
Control	1.09±0.035	0.37±0.008	0.23±0.007	12.87±0.381	1.63±0.680	355±12	1.90±0.458	0.71±0.004	0.66±0.017	0.07±0.019	0.27±0.004	0.26±0.059
Flooding-recovery	1.18±0.010	0.38±0.003	0.27±0.003	14.17±0.387	2.64±0.182	348±5	2.78±0.152	0.75±0.004	0.66±0.002	0.04±0.008	0.31±0.006	0.16±0.032
*P>Ff*	0.083	0.372	0.01	0.53	0.227	0.619	0.142	0.03	0.854	0.233	0.512	0.187

Ch *a*, chlorophyll a; Ch *b*, chlorophyll b; *A*, net photosynthetic rate; *g_s_*, stomatal conductance; *C_i_*, intercellular CO_2_ concentration; *E*, transpiration rate. Values are means ± SE (n = 6). The values followed by different letters significantly differed at P<0.05 according to Duncan's test. *P>Ff* indicate comparisons between treatments; *Ff*, effect of flooding stress.

**Figure 1:**
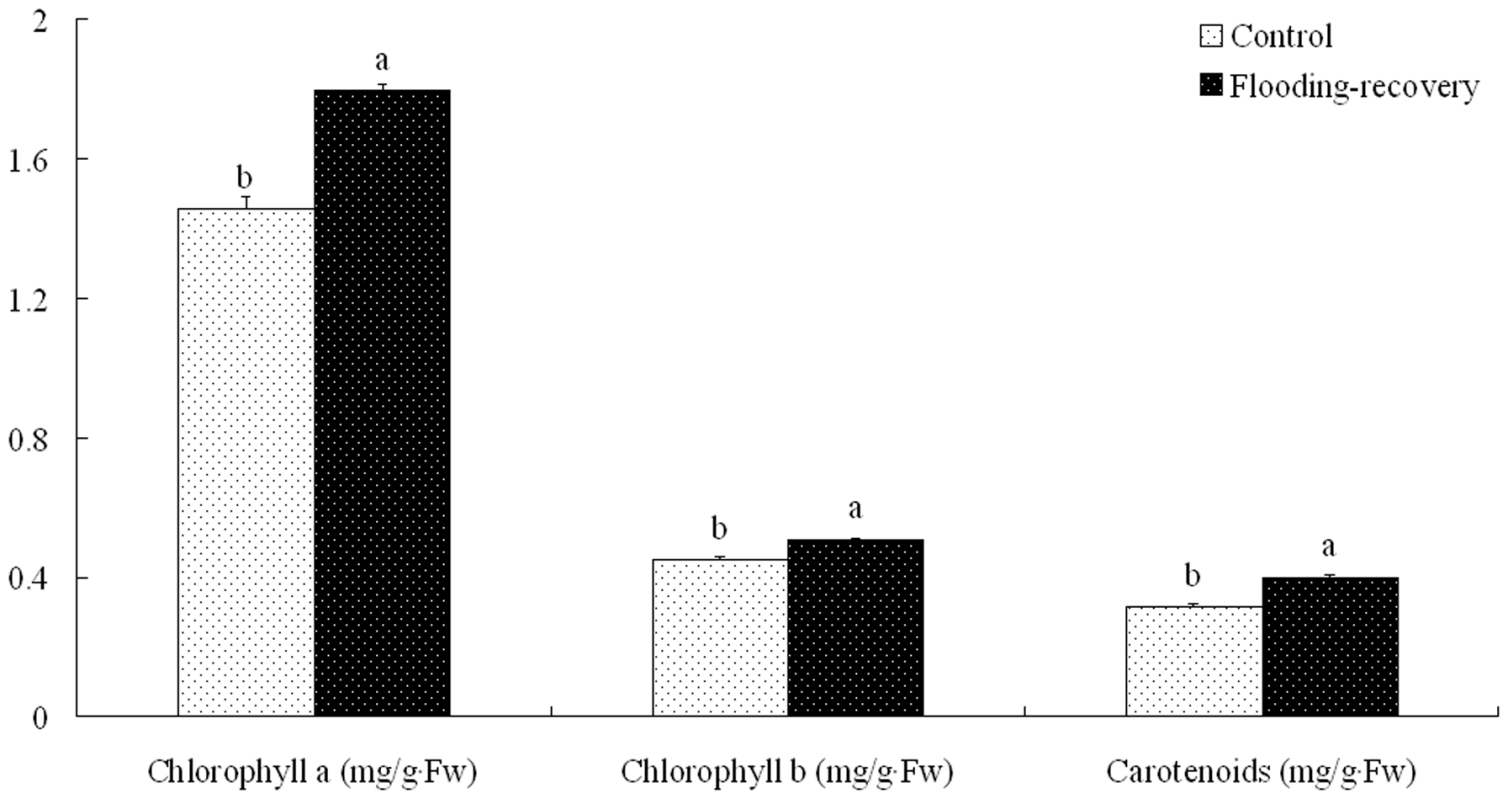
Comparable analyses of pigment contents in *L. chinense* during the recovery stage after winter flooding. Values are means ± SE (n = 6). The values followed by different letters significantly differed at P<0.05 according to Duncan's test.

**Figure 2:**
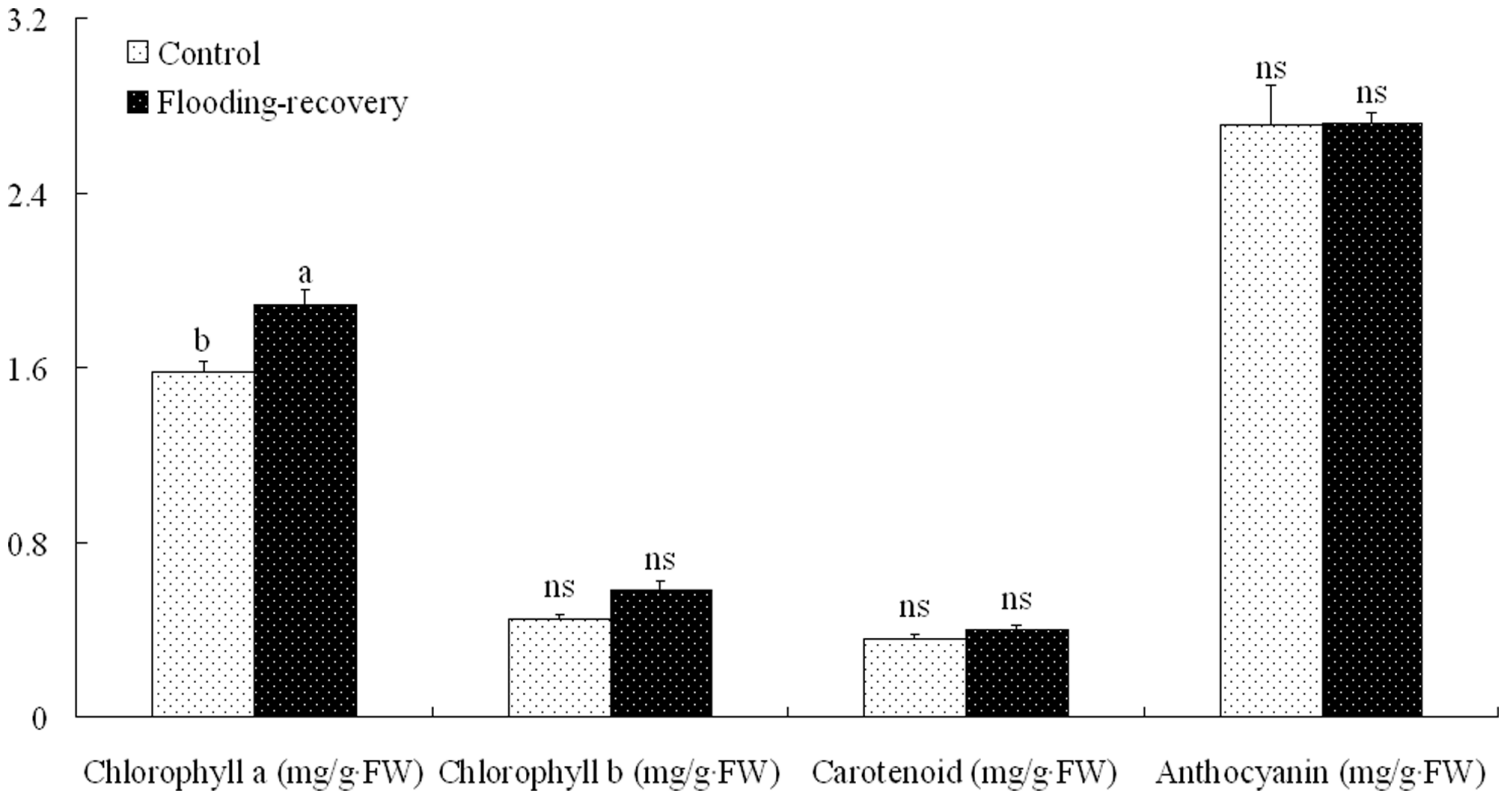
Comparable analyses of pigment contents in *S. variegata* during the recovery stage after winter floods. Values are means ± SE (*n* = 6). The values followed by different letters significantly differed at P<0.05 according to Duncan's test. ns, no significant differences.

During the recovery stage, the contents of Ch *a*, Ch *b*, and carotenoids in the submerged *L. chinense* were significantly higher than those in the controls (Fig. 1). The significant increases in the contents of Ch *a*, Ch *b*, carotenoids, *A*, *gs*, and *E* were also detected in the submerged *M. alba* compared with the controls. However, all chlorophyll fluorescence parameters were insignificantly affected by flooding stress (Table 7). The contents of Ch *a*, Ch *b*, carotenoids, and anthocyanin were higher in the submerged *S. variegata* than those in the controls, but significant increases were only found in Ch *a* (Fig. 2).

**Table 7:** Physiological responses of *M. alba* planted in the TGR WLFZ during the recovery stage after winter flooding.

	Ch *a* (mg/g Fw)	Ch *b* (mg/g Fw)	Carotenoids (mg/g Fw)	*A*	*g_s_*	*Ci*	*E*	Fv/Fm	Yield	Y(NPQ)	Y(NO)	qN
Control	2.08±0.058	0.63±0.021	0.49±0.015	11.90±1.021	0.67±0.082	357±15	2.81±0.530	0.70±0.010	0.68±0.005	0.02±0.007	0.30±0.011	0.09±0.032
Flooding-recovery	2.41±0.015	0.81±0.003	0.55±0.002	14.80±2.401	1.50±0.162	355±8	5.38±0.280	0.71±0.014	0.67±0.008	0.02±0.007	0.31±0.014	0.09±0.030
*P>Ff*	0.005	0.001	0.017	0.029	0.010	0.881	0.013	0.488	0.406	0.925	0.673	0.954

Ch *a*, chlorophyll a; Ch *b*, chlorophyll b; *A*, net photosynthetic rate; *g_s_*, stomatal conductance; *C_i_*, intercellular CO_2_ concentration; *E*, transpiration rate. Values are means ± SE (n = 6). The values followed by different letters significantly differed at P<0.05 according to Duncan's test. *P>Ff* indicate comparisons between treatments; *Ff*, effect of flooding stress.

## Discussion

As one of the largest reservoirs in the world, the TGR has a large area and a long riparian line of WLFZ. The total length of the WLFZ riparian line in the TGR is approximately 5711 km. The total area of the upper section (the zone of the elevation ranging from 170 m to 175 m) covers approximately 8000 ha, which is approximately 22% of the whole WLFZ area. However, the WLFZ is encountering several eco-environmental problems as described in the introduction. Therefore, the reasonable management and utilization of the TGR WLFZ has been a highly domestic and international concern. Forested riparian buffers can solve certain eco-environmental problems in the WLFZ, and maintain the ecological integrity of aquatic and terrestrial ecosystems. For example, Zhang *et al.* (2004) indicated that reforestation decreases surface runoff and soil erosion on severely eroded land. The annual total soil loss on the severely eroded bare land varied from 53 tons ha^−1^ to 256 tons ha^−1^. After reforestation, the annual soil erosion dramatically decreased to 2 tons ha^−1^ to 43 tons ha^−1^ from 1988 to 1990 and was negligible since then [27]. The white poplars planted in contaminated riparian forests could efficiently accumulate the Cd, Zn, and As of the soil [28]. Riparian forests could effectively reduce the nitrate and P concentrations in riparian soils by absorbing mineral N/P for plant growth. Wetlands dominated by plant communities remove N/P more effectively than non-vegetated wetlands because of the plant N/P uptake [29], [30]. However, the woody plants planted in the TGR WLFZ must endure winter flooding stress. Therefore, it is very important to screen winter flood-tolerant woody plant species suitable for the construction of riparian protection forests in the TGR WLFZ.

The new hydrological regimes of the TGR can dramatically influence riparian vegetation. About 405 vascular plant species belonging to 240 genera of 83 families were located in the riparian zone of TGRA in 2001 before the TGD impoundment. However, only 231 vascular plant species belonging to 169 genera of 61 families were distributed in the TGR WLFZ in 2009 [5], and only 127 vascular plant species were found in the TGR WLFZ in 2010 [6]. Therefore, hydrological alternation of the TGR caused a significant loss of vascular flora in the TGR WLFZ. Although 39 woody plant species were distributed in the TGR WLFZ, the survival of most of these woody plant species exists in a form of annual seedling and their appearances mainly depend on seed germination from the seed bank [31] without perennial roots. Thus, these species could not survive and recover during or after winter flooding. These woody plant species, in spite of belonging to perennial plant species, were not suitable for re-vegetation in the new riparian zone. Suitable woody plant species for the TGR WLFZ should be selected from flood-tolerant species by controlled experiments.

The survival rate analyses of 11 woody plant species after controlled winter flooding showed that all tested plants could endure long winter floods, thus suggesting that such plant species were suitable candidates for the construction of riparian protection forests in the TGR WLFZ. However, different flooding tolerances were found among the 11 woody plant species. In addition, other tested woody plant species such as *Lespedeza davidii* Franch., *Buxus ichangensis* Hatusima, and *Ficus tikoua* Bur. did not show strong flooding tolerance. The survival rate analyses suggested that the winter flooding tolerance of these plant species was affected by the submergence duration and depth. Thus, different shrub and arbor species should be reasonably distributed according to their flooding tolerance during re-vegetation activities.

The survival rate investigations of the six woody plants planted in the TGR WLFZ showed that these plants could survive and grow well after several winter flooding, thus suggesting that these plants were high-winter flood-tolerant species. Thus, the riparian protection forests in the TGR WLFZ could be popularized. However, the woody plant species planted at different altitudes within the TGR WLFZ must endure different winter flooding durations and depths, thus their reasonable distribution along the elevation of the WLFZ according to their flood tolerance should be concerned. The TGR WLFZ management must be enhanced during restoration, particularly for shrub species, which have slow growth rates. For example, many *L. Chinense*, *D. chinensis*, and *S. variegata* seedlings could survive during the primary recovery stage after winter floods, but parts of the surviving plants died annually because of the invasion of dominant weed communities.

Waterlogging and/or flooding stresses usually decreased chlorophyll pigment contents, photosynthetic efficiency, and PSII maximum efficiency and affect other chlorophyll fluorescence parameters (Yi et al. 2006; Li et al. 2011; Yang et al. 2011). In this study, the increases in the contents of chlorophyll pigments of *L. chinense*, *S. variegata*, *P.×canadensis* and *M. alba* plants, the insignificant variations in the chlorophyll fluorescence parameters of the submerged *P.×canadensis* and *M. alba* plants during the recovery stage after winter flooding stress suggested that such plant species have developed certain self-repairing capabilities during the recovery. The increases in chlorophyll pigments and photosynthesis in the submerged seedlings might efficiently benefit the total biomass accumulation and carbohydrate synthesis during the recovery. All these results suggested that these woody plant species could contribute to the construction of riparian protection forests in different elevations of the TGR WLFZ. In addition, the morphological, photosynthetic, and physiological responses of *P. stenoptera* [21], [22], *M. laxiflora* [23], and *D. chinense* [24] to flooding stress were also reported, which suggested these woody plant species could endure the flooding stress and recover well, and be used as candidate for the construction of riparian protection forests in the TGR WLFZ.

## Conclusions

Four experiments were used to determine the suitable woody species for the riparian protection forests in the TGR. The woody plant species survey in 2009 suggested that most of woody species existing as annual seedlings in the TGR WLFZ were unsuitable candidates for the riparian protection forests, whereas perennial *S. sebiferum*, *F. tikoua*, *L. chinense*, *M. alba*, *M. australis*, *P.×canadensis*, *S. matsudana*, *R. multiflora*, *S. variegata M. laxiflora*, and *D. chinense* could be found in the TGR WLFZ. In addition, survival rate analyses of the candidate woody plant species after controlled winter flooding showed arbor species such as *S. matsudana*, *P.×canadensis*, *M. alba*, *P. stenoptera*, *T. ascendens*, *M.glyptostroboides*, and shrub species such as *S. variegata*, *D. chinensis*, *L. chinense*, *M. laxiflora*, and *R. multiflora* could endure long winter floods, and should be used for the riparian protection forests in the TGR WLFZ. The survival rate investigations of the six woody plants planted in the TGR WLFZ showed that these plants could survive and grow well after several winter flooding. Thus, the riparian protection forests in the TGR WLFZ should be popularized. In addition, *P.×canadensis*, *M. alba*, *L. chinense*, and *S. variegata* could develop specific self-repairing mechanisms to stimulate biomass accumulation and carbohydrate synthesis via the increases in chlorophyll pigments and photosynthesis during recovery after winter floods. In addition, their reasonable distribution of these plant species along the elevation of the WLFZ and management should be considered during the construction of the riparian protection forests in the TGR WLFZ.
